# Impact of urine and mixed incontinence on long-term care preference: a vignette-survey study of community-dwelling older adults

**DOI:** 10.1186/s12877-020-1439-x

**Published:** 2020-02-18

**Authors:** Nicolas Carvalho, Sarah Fustinoni, Nazanin Abolhassani, Juan Manuel Blanco, Lionel Meylan, Brigitte Santos-Eggimann

**Affiliations:** University Center for Primary Care and Public Health (Unisanté), University of Lausanne, Route de la Corniche 10, CH-1010 Lausanne, Switzerland

**Keywords:** Long-term care, Older citizen opinion, Urine incontinence, Fecal incontinence

## Abstract

**Background:**

In view of population aging, a better knowledge of factors influencing the type of long-term care (LTC) among older adults is necessary. Previous studies reported a close relationship between incontinence and institutionalization, but little is known on opinions of older citizens regarding the most appropriate place of care. This study aimed at evaluating the impact of urine and/or fecal incontinence on preferences of community–dwelling older citizens.

**Methods:**

We derived data from the Lausanne cohort 65+, a population-based study of individuals aged from 68 to 82 years. A total of 2974 community-dwelling persons were interviewed in 2017 on the most appropriate place of LTC delivery for three vignettes displaying a fixed level of disability with varying degrees of incontinence (none, urinary, urinary and fecal). Multinomial logistic regression analyses explored the effect of respondents’ characteristics on their opinion according to Andersen’s model.

**Results:**

The level of incontinence described in vignettes strongly determined the likelihood of considering institutional care as most appropriate. Respondents’ characteristics such as age, gender, educational level, being a caregiver, knowledge of shelter housing or feeling supported by family influenced LTC choices. Self-reported incontinence and other indicators of respondents’ need, however, had no significant independent effect.

**Conclusion:**

Among older community-dwelling citizens, urinary and fecal incontinence play a decisive role in the perception of a need for institutionalization. Prevention and early initiation of support for sufferers may be a key to prevent this need and ensure familiar surrounding as long as possible.

## Background

Increasing public spending on long-term care (LTC) is a major economic issue that must be addressed [1]. Since the Switzerland monthly cost is 15 times higher per resident in nursing homes (NH) than for people staying at home [2], many authorities have implemented measures whose primary objective is to keep people at home as long as possible, while maintaining a good quality of life and ensuring access to appropriate care. As a result, over the last decade in Switzerland, the rate of long-term institutional care has fallen from 6.4 to 5.8% among those aged 65 and over and from 17.9 to 16.8% for those aged 80 and over [3]. However, a shift towards home care is not observed in the same proportions across Switzerland. There are still clear differences between French-speaking and German-speaking regions, the latter being characterized by a higher level of use of nursing homes and a lower level of home care [4]. Despite efforts to develop home care and support services, the demand for institutionalization is growing due to the increasing number of seniors and represents a significant challenge for the health system.

Causes for admission to NH are multiple and result from complex interactions between the person’s characteristics, caregivers, service providers and environment [5, 6]. According to Andersen’s model of access to care [7], factors involved in the choice between various options of LTC (e.g. delivered at home or in institutional setting) can be clustered into three broad categories: (1) predisposing factors representing socio-cultural characteristics of individuals prior to their illness; (2) enabling factors corresponding to logistical aspects of obtaining care and (3) need factors that represent functional and health problems.

Among need factors, incontinence has been identified as a potential predictor of institutionalization independently of other factors such as age, low self-rated health or functional impairments [8, 9]. However, these results were obtained in older patients while they entered in a NH, were partly inconsistent, and focused mainly on the presence of urinary disorders. Urinary incontinence (UI) and fecal incontinence (FI) are prevalent health conditions in old age and, although they may compromise the quality of life and increase caregivers’ burden [10, 11], they are often overlooked [12].

From a public health policy perspective, a better understanding of the role of UI and/or FI on older adults’ choices between LTC options, and of the factors associated with preferences, may help to identify targets for interventions to further reduce the share of institutionalization in LTC. The objective of the present study was to assess the impact of incontinence on LTC choices among community-dwelling older citizens. We hypothesized that: (i) UI and FI as components of needs for help and care both have an impact on the place of LTC considered as most appropriate and (ii) respondents’ predisposing, enabling and need factors, classified according to Andersen’s model, change LTC preferences.

## Methods

### Study population

Data on LTC choices were collected from January to April 2017 in the population of Lausanne (Switzerland), a city of 140,000 inhabitants. This study used a questionnaire on care mailed to all 3535 community-dwelling participants aged 68 to 82 years from the Lausanne cohort 65+ (Lc65+), a population-based study conducted on random samples drawn from the population register [13]. The response rate was 90.5% (*n* = 3195). Responses to the questionnaire on care were linked to the Lc65+ database providing additional information on participants’ characteristics collected at baseline (nationality, education) and in 2016. All information was self-reported. Prior to the data clean-up, 203 individuals (6.4% of observations) who had not answered questions relating to at least one of the three vignettes selected in this study were excluded and 18 were excluded for non-participation in 2016, leaving 2974 respondents for analysis. The study protocol was approved by the Vaud Ethics committee for human research (PB_2016–02506).

### Vignettes and LTC options

According to pre-tested methods [14], the questionnaire on care included a set of 10 vignettes displaying diverse needs for LTC, ordered by their level of severity. Of these, 3 vignettes presented a person with a same level of disability moderately affecting basic activities of daily living (moderate BADL), who lived with an able-bodied spouse. This fixed component included needs for help in preparing meals, housekeeping, shopping for groceries, getting out of bed in the morning, bathing and dressing, with preserved ability to get up from a chair and to walk inside. The 3 vignettes varied on continence status. The first (hereafter BADL only) was limited to the fixed component and did not mention continence problems. The second added the presence of UI (BADL+UI). The third added mixed (urinary and fecal) incontinence (BADL+MI). These vignettes specified that the person could not manage alone his or her incontinence.

After each vignette, the question “what arrangement do you think is the best” was asked, followed by the following possible responses: home (Home), sheltered house (SH), and nursing home (NH). The respondent’s choice was regarded as the dependent variable. The definition of SH provided within the survey questionnaire referred to a private apartment offering: 1) an adapted architecture, 2) an alarm system and 3) community spaces. Community health care centers (CHCC) or other home care organizations can supply assistance such as housework, meals at home and care.

### Anderson model factors

In accordance with Andersen’s model, respondents were assessed on three groups of independent variables: predisposing, enabling and need factors.

Predisposing factors included: *gender; age group* (68–72 / 73–77 / 78–82 years); *nationality* (Swiss / other / Swiss and other nationality); and *educational level* (compulsory schooling, corresponding to the International Standard Classification of Education ISCED 0–2 [15] / apprenticeship (ISCED 3) / baccalaureate (ISCED 4) / professional diploma (ISCED 6–7) / university or above (ISCED 8)).

Enabling factors included: *caregiver role* based on the question “Do you live with a person needing help?” (no / yes); *financial problems* based on a positive answer to the “Financial difficulties” item in a list of stressful life events experienced in the past 12 months; *anxiety* based on the question “During the past 4 weeks, have you often felt preoccupied and anxious? ” (no / yes); *depression* based on a reported medical diagnosis in the past 12 months or a positive response to either of the following two questions related to the past 4 weeks: “Have you often felt sad, depressed or discouraged? ” (no / yes) and “Have you often felt a lack of interest or pleasure in your usual activities? ” (no / yes); *isolation feelings* based on the question “During the past 4 weeks, how often did you feel isolated? ” (always, very often, often categorized into much / sometimes, rarely categorized into some / never); *household composition* based on the question “How many people do you live with?” followed by a list of cohabitants (categorized into living alone / with spouse, or with spouse and others categorized as with spouse / with others); *support from family* based on the question “With how many people in the family do you feel close enough to ask for help” (none labeled as no / one or more individuals labeled as yes); *potential informal care* assessed by the question “In case of long-term health problems, by whom could you possibly be helped? ” (spouse only / other family only / others / none / multiple responses); *knowledge of SH [*or *CHCC]* respectively evaluated by two similar questions “Do you know what a SH [CHCC] is, and what it can offer? ” (yes very well, rather yes categorized into yes / rather no, not at all categorized into no).

Five variables were considered as need factors: *cognitive difficulty* defined by any self-report of memory trouble affecting the daily life or difficulty concentrating selected in a list of troubles lasting 6 months or more; *mobility difficulty* based on a positive response to any of the following two questions “Do you have difficulty walking 100m [or climbing a flight of stairs without stopping] for health reasons? ” (none / some, much categorized into yes); *chronic diseases* defined by the number of reported conditions diagnosed by a physician, disturbing or treated in the past 12 months, selected in a list (hypertension, hypercholesterolemia, coronary artery disease, other cardiac disease, cerebrovascular disease, diabetes, chronic pulmonary disease, osteoporosis, arthritis and cancer) categorized into 0 / 1 / 2 and more conditions; *difficulties in activities of daily living (ADL)* based on reported difficulties or help in five instrumental activities (IADL) [16] and five basic activities (BADL) [17], categorized into no ADL difficulty / IADL difficulty only / BADL difficulty. *Incontinence* was defined by self-report of involuntary urine loss bothering since at least 6 months.

### Statistics

Friedman’s test was first used to check differences in the distribution of respondents’ preference for care options across the 3 vignettes. Post-hoc McNemar’s test was used for 2 × 2 analysis of differences.

In order to investigate the effect of respondents’ characteristics on their choices, Andersen’s model variables were screened for inclusion in multivariable regression models based on their bivariate association with the outcome.

Finally, we applied multinomial logistic regression models to predict LTC choices for each of the three vignettes separately, controlling for all Andersen’s model variables selected by bivariate analyses. For each vignette, SH was the base outcome. The relative risk ratio (RRR) Home vs SH indicates the effect of respondents’ characteristics on choices expressed among participants who selected either one of these two community-based options. Likewise, RRR NH vs SH describes the effects of these characteristics among those who selected either one of these two options implying a move from the usual home. We checked the variance inflation factor (VIF) and the tolerance as an indicator of multicollinearity. No collinearity between the variables was found, as the mean of VIF was less than 2 [18].

The significance alpha level was fixed to 0.05. All computations were performed using Stata Software release 15.1 (StataCorp, College Station, TX).

## Results

### Profile of participants

Table 1 shows the characteristics of the sample. The majority of respondents were female (58.7%), 41.7% were aged between 69 and 73 years and 90.2% were Swiss. Education was limited to compulsory schooling for 15.5% of participants, 36.5 reported university or professional degrees and 48.0% had completed an intermediate level. 9.6% of participants reported difficulties in IADL only and 14.8% in BADL. Incontinence was mentioned by 13.9% of respondents. Out of 2974 survey participants, 96.2% responded for the BADL only vignette, 97.7% for the BADL+UI vignette and 97.1% for the BADL+MI vignette.

**Table 1:** Descriptive characteristics of survey participants (*n* = 2974) according to Andersen’s model^a^

	N (%)
Predisposing factors	
Gender	
Men	1229 (41.3)
Women	1745 (58.7)
Age group	
68–72	1239 (41.7)
73–77	964 (32.4)
78–82	771 (25.9)
Nationality	
Swiss	2303 (77.6)
Other	290 (9.8)
Swiss and other nationality	373 (12.6)
Educational level	
Compulsory schooling (ISCED 0–2)	460 (15.5)
Apprenticeship (ISCED 3)	1176 (39.6)
Baccalaureate (ISCED 4)	250 (8.4)
Professional diploma (ISCED 6–7)	506 (17.0)
University or above (ISCED 8)	579 (19.5)
Enabling factors	
Caregiver role	
Yes	211 (7.2)
Financial problems	
Yes	179 (6.1)
Knowledge of SH	
Yes	2422 (82.9)
Knowledge of CHCC	
Yes	2456 (84.3)
Anxiety	
Yes	790 (26.7)
Depression	
Yes	721 (24.5)
Isolation feelings	
Never	1416 (47.7)
Some	1344 (45.3)
Much	209 (7.0)
Household composition	
Alone	1178 (40.0)
With spouse	1684 (57.1)
With others	86 (2.9)
Support from family	
Yes	2647 (90.1)
Potential informal care	
Spouse only	710 (24.1)
Other family only	445 (15.1)
Others	253 (8.6)
None	331 (11.2)
Multiple responses	1209 (41.0)
Need factors	
Cognitive difficulty	
Yes	404 (13.7)
Incontinence	
Yes	410 (13.9)
Chronic disease (s)	
0	918 (31.)
1	924 (31.2)
2 or more	1117 (37.8)
Mobility difficulty	
Yes	514 (17.5)
ADL limitation	
No ADL difficulty	2223 (75.7)
IADL difficulty only	281 (9.6)
BADL difficulty	434 (14.8)

Place of LTC delivery: *SH* sheltered house, *NH*, nursing home

Respondents’ characteristics: *ISCED* international standard classification of education, *CHCC* community health care center, *ADL* activities of daily living, *IADL* instrumental activities of daily living, *BADL* basic activities of daily living, *UI* urine incontinence, *MI* mixed (urine and fecal) incontinence

^a^Andersen, R. M. (1995). Revisiting the behavioral model and access to medical care: does it matter? J Health Soc Behav, 36(1), 1–10

### Effect of incontinence displayed in the vignette

**Fig. 1:**
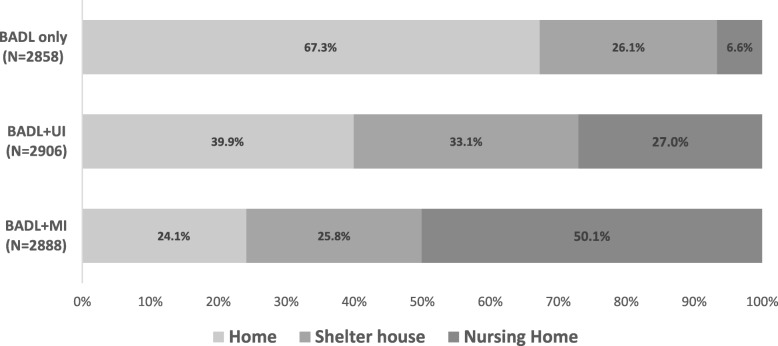
Older citizens’ opinion on the most appropriate place of long-term care delivery expressed for 3 vignettes varying on continence status. The 3 vignettes presented a person with a same level of disability moderately affecting basic activities of daily living (moderate BADL), who lived with an able-bodied spouse. This fixed component included needs for help in preparing meals, housekeeping, shopping for groceries, getting out of bed in the morning, bathing and dressing, with preserved ability to get up from a chair and to walk inside. The 3 vignettes varied on continence. The first (hereafter BADL only) was limited to the fixed component and did not mention continence problems. The second added the presence of urine incontinence (BADL+UI). The third added mixed (urinary and fecal) incontinence (BADL+MI). Information given in the vignette specified that the disabled person could not manage alone his or her incontinence and lives with an able-bodied spouse. There was a significant difference in LTC choices depending on incontinence severity displayed in the vignette (Friedman test, *p* < 0.001)

There was a significant difference in LTC choices depending on incontinence severity displayed in the vignette (Friedman test, *p* < 0.001) (Fig. 1). Post hoc analysis with McNemar test revealed that the proportion of persons who chose Home decreased significantly (from 67.3 to 24.1%, p < 0.001) and the proportion of people who chose NH increased significantly (from 6.6 to 50.1%, p < 0.001) from the BADL only vignette to the BADL+MI vignette. The proportion of people choosing SH did no change significantly between the 3 vignette, SH option was selected by 26% of the participants for the first and the third vignette and by 33.1% for the intermediate (BADL+UI).

### Effect of respondents’ characteristics

Table 2 shows that in all subgroups defined by respondents’ own characteristics, an absolute majority selected the Home option for the BADL only vignette while the most frequent choice, if not always reaching absolute majority, was NH for the BADL+MI vignette. However, Table 2 also displays bivariate differences among older people choosing Home, SH or NH. Gender and educational level were predisposing factors related to choices for all 3 vignettes while age influenced opinions only for the two vignettes mentioning incontinence. Caregiver role, household composition, and potential informal care were selected as enabling factors in analyses for the 3 vignettes. Knowledge of SH was a significant enabling factor for the two vignettes mentioning incontinence, depression and support from family for the BADL only vignette, financial problems for the BADL and BADL+MI vignettes and knowledge of CHCC for the BADL+MI vignette. Among needs factors, respondents’ self-reported cognitive and mobility difficulties were significantly associated with their LTC choices for the BADL+MI vignette. The number of chronic diseases, ADL difficulties or UI self-reported by respondents had no influence on their opinions for the three vignettes.

**Table 2:** Effect of older adults’ characteristics on their opinion regarding the most appropriate long-term care option (Home, sheltered house (SH) or nursing home (NH)) expressed for 3 vignettes: bivariate analysis

	BADL (*N* = 2861)					BADL+UI (*N* = 2907)					BADL+MI (*N* = 2888)				
	N	Home (1925)	SH (747)	NH (189)	p	N	Home (1161)	SH (961)	NH (785)	p	N	Home (697)	SH (745)	NH (1446)	p
		%	%	%			%	%	%			%	%	%	
Predisposing factors															
Gender	2861					2907					2888				
Men	1168	72.3	21.7	6.1	0.000	1201	46.2	31.0	22.8	0.000	1195	28.5	26.4	45.0	0.000
Women	1693	63.9	29.2	7.0		1706	35.5	34.5	30.0		1693	21.0	25.3	53.6	
Age group	2861					2907					2888				
68–72	1194	67.8	26.0	6.2	0.856	1210	38.2	35.6	26.2	0.006	1206	20.2	27.8	52.0	0.000
73–77	932	67.2	26.4	6.4		955	39.3	34.1	26.6		942	24.1	24.2	51.7	
78–82	735	66.7	25.9	7.5		742	43.7	27.5	28.8		740	30.5	24.6	44.9	
Nationality	2854					2899					2880				
Swiss	2223	68.0	25.6	6.5	0.301	2252	39.2	32.7	28.1	0.090	2239	23.6	25.0	51.4	0.067
Other	277	62.8	28.5	8.7		284	45.4	32.8	21.8		279	28.3	28.3	43.4	
Swiss and other nationality	354	66.1	28.2	5.7		363	39.7	35.8	24.5		362	23.8	29.0	47.2	
Educational level	2860					2904					2885				
Compulsory schooling (ISCED 0–2)	434	65.2	24.0	10.8	0.002	442	41.0	29.0	30.1	0.004	439	31.2	23.7	45.1	0.003
Apprenticeship (ISCED 3)	1128	65.6	27.7	6.7		1147	40.3	31.0	28.8		1134	24.8	23.9	51.3	
Baccalaureate (ISCED 4)	241	65.6	30.7	3.7		248	32.7	43.2	24.2		242	19.8	26.9	53.3	
Professional diploma (ISCED 6–7)	493	70.2	24.3	5.5		496	41.3	34.5	24.2		497	21.7	28.4	49.9	
University or above (ISCED 8)	564	70.6	24.1	5.3		571	40.3	35.0	24.7		573	21.1	28.6	50.3	
Enabling factors															
Caregiver role	2820					2861					2843				
No	2612	66.7	26.6	6.7	0.032	2655	39.2	33.6	27.2	0.012	2639	23.1	26.0	50.9	0.000
Yes	208	75.5	19.2	5.3		206	49.0	25.2	25.7		204	36.8	22.6	40.7	
Financial problems	2843					2890					2871				
No	2676	67.8	25.9	6.4	0.039	2720	39.5	33.3	27.2	0.489	2701	23.4	26.0	50.6	0.014
Yes	167	59.3	30.5	10.2		170	44.1	31.2	24.7		170	32.9	25.3	41.8	
Enabling factors															
Knowledge of SH	2817					2862					2841				
No	475	69.7	23.4	6.9	0.294	485	44.3	33.6	22.1	0.014	485	32.2	21.4	46.4	0.000
Yes	2342	66.7	26.8	6.4		2377	38.8	33.1	28.1		2356	21.8	26.8	51.4	
Knowledge of CHCC	2806					2853					2831				
No	427	68.6	25.1	6.3	0.789	441	41.7	34.7	23.6	0.179	435	32.4	22.3	45.3	0.000
Yes	2379	66.9	26.4	6.6		2412	39.3	32.8	27.9		2396	22.0	26.4	51.7	
Anxiety	2841					2887					2869				
No	2085	67.9	25.7	6.5	0.684	2121	39.4	33.5	27.1	0.592	2105	23.4	26.0	50.6	0.216
Yes	756	66.1	27.1	6.8		766	41.5	32.1	26.4		764	26.6	25.3	48.2	
Depression	2835					2880					2861				
No	2148	68.7	25.1	6.2	0.013	2186	40.0	33.3	26.7	0.834	2175	24.4	25.7	50.1	0.941
Yes	687	62.7	29.3	8.0		694	39.1	33.1	27.8		686	23.8	26.1	50.2	
Isolation feelings	2856					2902					2883				
Never	1372	69.3	24.7	6.0	0.167	1387	40.3	32.5	27.2	0.812	1377	22.7	25.1	52.2	0.064
Some	1293	64.9	28.0	7.1		1316	39.3	33.4	27.4		1308	25.1	25.9	49.0	
Much	190	68.4	24.2	7.4		199	41.7	34.7	23.6		198	28.8	29.3	41.9	
Household composition	2841					2884					2863				
Alone	1130	60.8	29.9	9.3	0.000	1152	31.9	34.9	33.2	0.000	1137	19.8	25.0	55.2	0.000
With spouse	1630	72.3	23.0	4.7		1646	45.7	31.5	22.8		1642	27.2	26.4	46.4	
With others	81	60.5	34.6	4.9		86	36.1	39.5	24.4		84	21.4	27.4	51.2	
Support from family	2824					2870					2851				
No	277	62.8	24.2	13.0	0.000	279	38.4	33.7	28.0	0.869	272	29.4	23.9	46.7	0.086
Yes	2547	67.8	26.2	5.9		2591	39.9	33.2	26.9		2579	23.4	26.1	50.5	
Enabling factors															
Potential informal care	2836					2882					2863				
Spouse only	676	71.0	24.1	4.9	0.000	690	48.7	30.1	21.2	0.000	690	30.6	24.9	44.5	0.000
Other family only	419	60.4	32.5	7.2		435	33.6	35.4	31.0		431	24.8	24.8	50.4	
Others	242	63.6	26.0	10.3		246	36.2	33.7	30.1		239	21.3	23.0	55.7	
None	326	59.2	30.4	10.4		322	28.0	37.0	35.1		317	16.4	25.2	58.4	
Multiple responses	1173	71.2	23.4	5.5		1189	41.6	32.3	26.1		1186	22.9	27.3	49.8	
Need factors															
Cognitive difficulty	2837					2883					2865				
No	2458	67.6	25.8	6.7	0.783	2494	39.6	33.4	27.0	0.399	2477	23.2	25.7	51.1	0.017
Yes	379	66.0	27.4	6.6		389	42.4	30.1	27.5		388	29.6	25.3	45.1	
Incontinence	2837					2883					2865				
No	2450	67.6	25.8	6.6	0.738	2485	40.3	32.8	26.9	0.660	2467	24.4	25.5	50.1	0.673
Yes	387	65.6	27.4	7.0		398	37.9	33.7	28.4		398	22.4	26.6	51.0	
Chronic disease (s)	2846					2892					2873				
0	887	69.1	24.9	6.0	0.347	896	39.4	32.4	28.2	0.821	894	23.0	26.7	50.2	0.647
1	883	65.0	27.3	7.7		902	40.5	32.7	26.8		894	23.4	26.4	50.2	
2 and more	1076	67.5	26.3	6.2		1094	39.7	34.2	26.1		1085	25.5	24.7	49.8	
Mobility difficulty	2817					2866					2847				
No	2342	67.9	25.8	6.3	0.192	2372	39.9	33.3	26.9	0.883	2355	22.8	26.2	50.9	0.001
Yes	475	64.4	27.4	8.2		494	41.1	32.6	26.3		492	30.7	23.8	45.5	
ADL limitation	2825					2873					2855				
No ADL difficulty	2149	68.1	25.5	6.4	0.398	2185	40.1	33.2	26.7	0.870	2170	23.0	26.1	50.9	0.124
IADL difficulty only	274	63.9	27.4	8.8		270	37.8	33.3	28.9		266	25.9	23.3	50.8	
BADL difficulty	402	65.4	28.1	6.5		418	41.4	31.6	27.0		419	28.4	25.8	45.8	

The 3 vignettes presented a person with a same level of disability moderately affecting basic activities of daily living (moderate BADL), who lived with an able-bodied spouse. This fixed component included needs for help in preparing meals, housekeeping, shopping for groceries, getting out of bed in the morning, bathing and dressing, with preserved ability to get up from a chair and to walk inside. The 3 vignettes varied on continence. The first (hereafter BADL only) was limited to the fixed component and did not mention continence problems. The second added the presence of urine incontinence (BADL + UI). The third added mixed (urinary and fecal) incontinence (BADL + MI). Information given in the vignette specified that the disabled person could not manage alone his or her incontinence and lives with an able-bodied spouse

Place of LTC delivery: *SH* sheltered house, *NH* nursing home

Repondants’ characteristics: *ISCED* international standard classification of education, *CHCC* community health care center, *ADL* activities of daily living, *IADL* instrumental activities of daily living, *BADL* basic activities of daily living, *UI* urine incontinence, *MI* mixed incontinence

Table 3 reports relative risk ratios for Andersen’s model factors included in separate multinomial logistic regressions corresponding to the three vignettes.

**Table 3:** Effect of older adults’ characteristics on their opinion regarding the most appropriate long-term care option (Home, sheltered house (SH) or nursing home (NH)) expressed for 3 vignettes: multinomial logit regressions

	BADL *N* = 2703				BADL+UI *N* = 2781				BADL+MI *N* = 2668			
	Home vs SH		NH vs SH		Home vs SH		NH vs SH		Home vs SH		NH vs SH	
	RRR	95 CI%	RRR	95 CI%	RRR	95 CI%	RRR	95 CI%	RRR	95 CI%	RRR	95 CI%
Predisposing factors												
Gender												
(Men)												
Women	0.74**	0.60–0.90	0.65*	0.45–0.96	0.80*	0.65–0.97	1.02	0.82–1.27	0.86	0.67–1.10	1.16	0.94–1.42
Age group												
(68–72)												
73–77					1.11	0.90–1.36	1.05	0.83–1.31	1.44**	1.11–1.87	1.14	0.92–1.41
78–82					1.41**	1.12–1.78	1.31*	1.02–1.68	1.63**	1.23–2.16	0.93	0.73–1.18
Educational level												
(Compulsory schooling) (ISCED 0–2)												
Apprenticeship (ISCED 3)	0.72*	0.54–0.96	0.50**	0.32–0.80	0.80	0.60–1.06	0.84	0.62–1.14	0.81	0.58–1.13	1.08	0.80–1.46
Baccalaureate (ISCED 4)	0.76	0.52–1.11	0.28**	0.12–0.61	0.50***	0.34–0.74	0.51**	0.34–0.76	0.63	0.39–1.02	0.95	0.63–1.41
Professional diploma (ISCED 6–7)	0.88	0.63–1.22	0.46**	0.26–0.82	0.70*	0.51–0.96	0.64*	0.45–0.91	0.59**	0.40–0.88	0.91	0.65–1.28
University or above (ISCED 8)	0.80	0.58–1.11	0.43**	0.24–0.76	0.71*	0.51–0.97	0.70*	0.49–0.99	0.64*	0.44–0.95	0.95	0.68–1.32
Enabling factors												
Caregiver role												
(No)												
Yes	1.43	0.97–2.10	1.42	0.68–2.96	1.61*	1.11–2.34	1.43	0.93–2.18	1.58*	1.03–2.42	0.99	0.67–1.47
Financial problems												
(No)												
Yes	0.97	0.66–1.43	1.45	0.78–2.69					1.42	0.90–2.24	0.89	0.59–1.35
Depression												
(No)												
Yes	0.84	0.68–1.03	0.97	0.67–1.43								
Knowledge of SH												
(No)												
Yes					0.94	0.75–1.19	1.30	0.98–1.71	0.70*	0.50–0.98	0.84	0.62–1.13
Knowledge of CHCC												
(No)												
Yes									0.67*	0.47–0.96	0.96	0.70–1.32
Enabling factors												
Household composition												
(Alone)												
With spouse	1.26	0.97–1.63	0.58*	0.35–0.95	1.11	0.85–1.43	0.71*	0.54–0.95	1.12	0.81–1.56	0.86	0.65–1.12
With Others	0.89	0.53–1.49	0.50	0.17–1.52	0.91	0.54–1.54	0.56	0.31–1.02	1.02	0.49–2.09	0.97	0.54–1.75
Potential informal care												
(Spouse only)												
Other family only	0.92	0.64–1.31	0.87	0.43–1.75	0.66*	0.46–0.95	0.88	0.59–1.31	0.82	0.53–1.29	0.96	0.65–1.40
Others	1.24	0.81–1.90	1.50	0.71–3.17	0.82	0.54–1.25	0.97	0.62–1.53	0.96	0.56–1.63	1.15	0.74–1.79
None	0.97	0.66–1.42	1.14	0.56–2.32	0.56*	0.38–0.84	1.01	0.67–1.53	0.57*	0.35–0.95	1.13	0.76–1.69
Multiples responses	1.19	0.93–1.52	1.17	0.70–1.95	0.88	0.70–1.11	1.04	0.79–1.37	0.79	0.59–1.05	0.98	0.76–1.26
Support from family												
(No)												
Yes	0.94	0.68–1.31	0.56*	0.34–0.93								
Need factors												
Cognitive difficulty												
(No)												
Yes									1.21	0.88–1.66	0.90	0.68–1.19
Mobility difficulty												
(No)												
Yes									1.22	0.90–1.65	0.98	0.76–1.28

Legend Table 3: The 3 vignettes presented a person with a same level of disability moderately affecting basic activities of daily living (moderate BADL), who lived with an able-bodied spouse. This fixed component included needs for help in preparing meals, housekeeping, shopping for groceries, getting out of bed in the morning, bathing and dressing, with preserved ability to get up from a chair and to walk inside. The 3 vignettes varied on continence. The first (hereafter BADL only) was limited to the fixed component and did not mention continence problems. The second added the presence of urine incontinence (BADL + UI). The third added mixed (urinary and fecal) incontinence (BADL + MI). Information given in the vignette specified that the disabled person could not manage alone his or her incontinence and lives with an able-bodied spouse

For each vignette, SH was the base outcome

Place of LTC delivery: SH, sheltered house; NH, nursing home

Respondents’ characteristics: *ISCED* international standard classification of education, *CHCC* community health care center, *ADL* activities of daily living, *IADL* instrumental activities of daily living, *BADL* basic activities of daily living, *UI* urine incontinence, *MI* mixed incontinence

*RRR* relative risk ratio, *95 CI%* 95 confidence interval;

() refers to the base

*p < .05

**p < .01

***p < .001

### Predisposing factors

#### Home vs SH

Among respondents who selected one of the two community-based options (Home or SH), women were less likely than men to choose Home for both the BADL only and the BADL+UI vignettes. Participants reporting apprenticeship were also less likely to choose Home for the BADL only vignette than those with education limited to compulsory schooling, as did participants with baccalaureate, professional diploma or higher educational level for the BADL+UI vignette, and participants with professional diploma or higher educational level for the BADL+MI vignette. By contrast, older participants chose Home more frequently than the youngest for the BADL+UI and the BADL+MI vignettes.

#### NH vs SH

Among respondents who did not consider Home as the most appropriate option (i.e. choosing either SH or NH), women and those with more than compulsory schooling were less likely to choose NH than men and respondents with the lowest level of education for the BADL only vignette. Participants with higher levels of education (baccalaureate, professional diploma or higher) were also less likely to choose NH for the BADL+UI vignette than respondents with education limited to compulsory schooling, while the oldest preferred the NH option more often than the younger in this case.

### Enabling factor

#### Home vs SH

Among respondents who selected one of the two community-based options, caregivers were more likely to choose Home than those who did not report a caregiver’s role both for the BADL+UI and the BADL+MI vignettes. By contrast, for these two vignettes, participants with poor perspective of receiving informal care chose SH more than those expecting help from a spouse in case of need. For the BADL+MI vignette, participants reporting a good knowledge of SH or CHCC privileged SH more than those feeling uninformed.

#### NH vs SH

Among respondents who did not considered Home as the most appropriate option, those living with a spouse were less likely than those living alone to choose NH for the BADL only and the BADL+UI vignettes. Participants feeling supported by their family selected NH for the BADL only vignette less frequently than those who reported no family support.

### Need factors

None of the tested respondents’ need factors had significant influence on LTC preferences for any of the three vignettes in multivariate analyses once predisposing and enabling factors were controlled.

## Discussion

In this population-based study of people aged between 68 and 82 years, we found that (i) incontinence as part of the disability profile, and its severity, significantly influenced the opinion expressed by older citizens regarding the place most appropriate for LTC delivery; (ii) the own characteristics of respondents such as age, gender, education level, caregiver role and knowledge of community-based services had a significant impact on LTC choices while their need characteristics, including self-reported difficulties in ADL or incontinence, did not influence opinions.

The significant influence of UI, shifting preferences towards SH and NH, is consistent with results of previous studies that reported an increased risk of admission to NH in people suffering from UI [19, 20] attributed to its physical and psychosocial consequences [21]. In case of MI, institutionalization was the most frequent choice in our population. This observation suggests that as the severity of incontinence increases, so does the risk of NH admission. However, other studies reported an absence of interaction between UI and FI on the prediction of institutionalization [22, 23]. Discrepancies may stem from methodological differences as we used multivariate analysis while other studies have applied univariate analysis [23, 24]. Moreover, the lack of distinction between UI and FI [20] and absence of consensus on the definition of FI may explain different effects of FI on institutionalization [24].

Surprisingly, personal need characteristics of older citizens, especially their own ADL disability and incontinence, had no significant impact on their opinions regarding LTC. The similarity of opinions of those without incontinence, and those with incontinence suggest that normalization, living with the condition and managing it ceases to become relevant as opposed to conjectural future incontinence. However, LTC preferences were affected by demographic and socio-economic factors. Women were more likely to choose institutionalization when the vignette presented UI. As primary caregivers at home [25], they could be more conscious of the workload imputable to the UI disability and therefore choose institutionalization more than men. UI may also have a greater impact on men’s general health [21] and functional capacities [26]. With the addition of FI to the disability profile, the difference between men and women in LTC choices receded and NH was advocated by both genders. In line with this observation, several studies have found that the association of UI and FI was not a gender-specific predictor of institutionalization [23, 27]. The citizens’ age also influenced choices. While LTC delivered at Home was still preferred in all age groups in case of UI, older respondents selected this option more often. With MI, NH was the most frequent choice irrespective of age but older people were also more likely to prefer the Home option than younger participants. However, previous studies investigating the use of LTC did not show any interaction between age and UI or FI on NH entry [9, 24]. Educational level was the third predisposing factor weighing on choices. Respondents with higher levels of education were more likely to choose SH rather than NH when the vignette presented a person with UI. They may have better knowledge of disabilities, treatments and the range of coping solutions. In a systematic review, Luppa et al. [28] reported some impact of education on LTC choice with preference for institutionalization associated to a low level of education.

Enabling factors may influence older citizens’ opinions on appropriate LTC. When the vignette showed a person with UI, respondents who were caregivers recommended Home more often than those who were not. However, Thomas [29] and Di Rosa [30] reported that incontinence was the most frequent complaint and source of stress for caregivers. Several studies revealed that between 36 and 53% of caregivers reported burden caused by UI and more for MI [24, 31–33]. Moreover, many studies indicated that caregivers’ burden was a predictor of institutionalization [34–36]. Severe incontinence may exceed the potential of informal care and Kauppi et al. [37] have reported that partial assistance provided for older person (i.e., covering only part of the gap to reduce but not eliminate the excessive burden) was a predictor of institutionalization. The knowledge of health services also influenced LTC choices, particularly in the case of severe incontinence. Independently of the definition of SH provided in the study questionnaire, previous knowledge regarding SH or CHCC was associated with a more frequent choice of the SH option, both when the majority of the respondents selected LTC in the community as well as when care at home was no more considered as appropriate. The amount and accessibility of information had an impact on LTC use described in another study [38]. Strain et al. [39] also reported that the reason why caregivers did not use alternatives to institutionalization was their lack of knowledge of day center, day hospital and home respite service. However, SH as an intermediate structure between home care and institutionalization has not been well investigated in the literature. Proposing different structures as an alternative to NH admissions could permit to distinguish more precisely the factors related to the choice of LTC [40].

### Strengths and limitations

An important strength of our research was the use of a set of vignettes with a variable component (continence status) on a comparable base (ADL), applied to a population-based cohort of randomly selected older citizens. This allowed to assess the specific effect of incontinence on older persons opinions, as citizens, regarding appropriate LTC. Moreover, the influence of personal characteristics on citizens’ opinions could be investigated using detailed individual data. Nevertheless, the information provided to the survey participants did not specify the type of urine incontinence, while it can take several forms such as stress incontinence, urge incontinence or a combination of the both [21]. It would also be interesting to consider the frequency and intensity of incontinence [9]. However, incontinence was described in the vignette as a problem generating a need for help. As a respondent’s characteristic, UI was self-reported, and therefore may be underestimated. Its severity was not quantified and no question was asked on FI. Finally, survey participants expressed their citizen’s opinions on abstract situations. Their personal choices might be different when facing themselves the exact circumstances presented in the vignettes.

## Conclusion

Our results suggest that older citizens’ opinions regarding the most appropriate LTC options are mainly influenced by their socio-structural and economic characteristics, and are largely independent of their own health. Overall, individuals characteristics do not seem to have a strong influence on citizens’ opinions. The effect of incontinence, and particularly of MI, on the acceptance of home care must be considered by policy makers. Indeed, our findings point to the necessity of considering the burden of incontinence acting as a barrier to maintain older adults in the community. Possible actions to promote further developments of LTC provision in the community may include preventive measures targeting risk factors for UI and FI along the life course, appropriate medical care of their causes and manifestations, and increased caregivers’ support, particularly in groups at risk (i.e. with lower socio-economic resources) of turning to LTC provided in an institutional setting.

## Data Availability

The authors do not have permission to share data.

## Abbreviations

ADL: Activities of daily living
BADL: Basic activities of daily living
CHCC: Community health care centers
FI: Fecal incontinence
H: Usual home
IADL: Instrumental activities of daily living
ISCED: International Standard Classification of Education
LC65 +: Lausanne cohort 65+
LTC: Long-term care
NH: Nursing home
RRR: Relative risk ratio
SH: Sheltered home
UI: Urinary incontinence
VIF: Variance inflation factor
